# Supplementary material for: A Novel Technique to Characterize Klebsiella pneumoniae Populations Indicates that Mono-Colonization is Associated with Risk of Infection
Source: bioRxiv. 2025 Nov 13:2025.11.05.686704. Preprint. [Version 2] doi: 10.1101/2025.11.05.686704 (PMC12642541; doi:10.1101/2025.11.05.686704)
Supplement: 1 [file NIHPP2025.11.05.686704V2-supplement-1.pdf]

bioRxiv preprint doi: https://doi.org/10.1101/2025.11.05.686704; this version posted November 13, 2025. The copyright holder for this preprint (which was not certified by peer review) is the author/funder, who has granted bioRxiv a license to display the preprint in perpetuity. It is made available under aCC-BY 4.0 International license.

Supplemental Figures

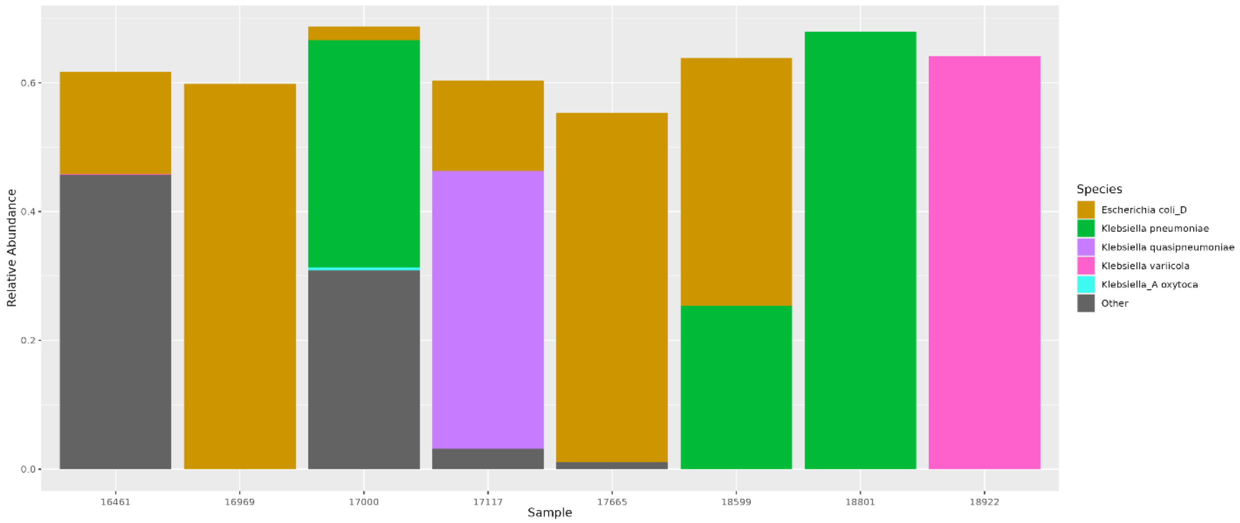

Supplemental Figure 1: Species composition based on Illumina Sequencing of *Klebsiella* Mixed and Mono-Colonized Rectal Swabs used for validation.

Eight glycerol amended swab samples, four mixed and four mono-colonized based on analysis above, were struck out on MacConkey agar to select for Gram-negative and then DNA was extracted. Samples were subjected to Illumina sequencing and bacterial species composition was determined by InStrain.

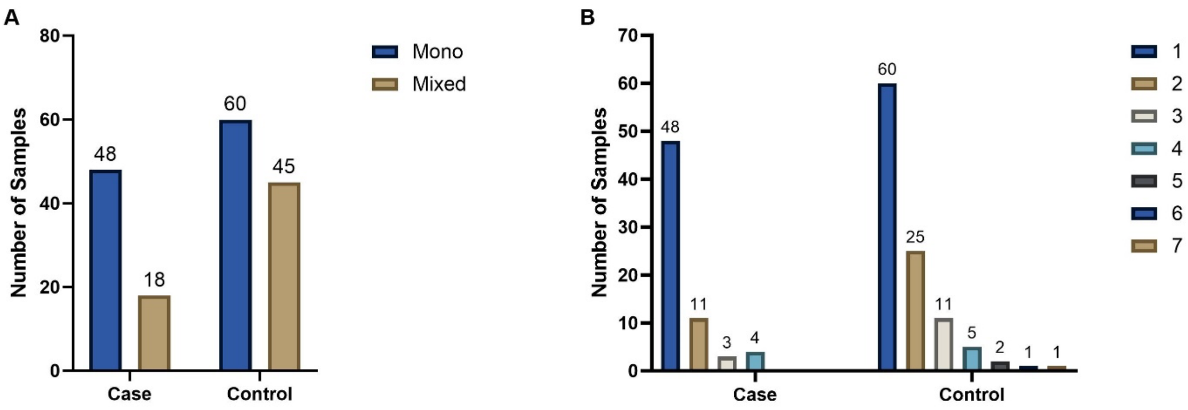

Supplemental Figure 2: Mono- and mixed colonization in cases of *Klebsiella* infection and controls

Colonization and case status of all patients were evaluated based on mono- or mixed colonization status (A) or the number of *wzi* types present in each sample (B).

bioRxiv preprint doi: https://doi.org/10.1101/2025.11.05.686704; this version posted November 13, 2025. The copyright holder for this preprint (which was not certified by peer review) is the author/funder, who has granted bioRxiv a license to display the preprint in perpetuity. It is made available under aCC-BY 4.0 International license.

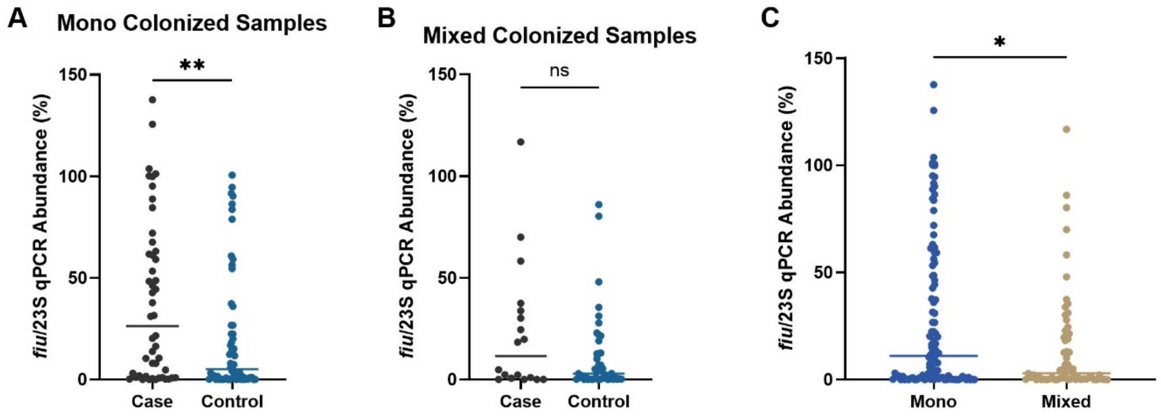

Supplemental Figure 3: Mono-colonized case samples have significantly higher *Klebsiella* relative abundance than controls

(A-C) The *Klebsiella* relative abundance in each rectal swab was quantified using a validated qPCR assay quantifying the conserved *Klebsiella fiu* gene and the panbacterial 23S rRNA gene. The ratio of the *fiu* and 23S rRNA gene were used to calculate the *Klebsiella* relative abundance in case or control patient rectal swabs from mono- (A) or mixed (B) colonized patients. The *Klebsiella* relative abundance calculated previously (11) was also compared between mono- and mixed colonized patients (C). For (A-B), **, $p < 0.001$ using a Mann-Whitney t-test with $n \geq 18$. For (C), *, $p < 0.05$ with an $n=171$ and Mann-Whitney t-test.

Supplemental Tables

Supplemental Table 1: Applying *wzi*-Seq to a single *Klebsiella* strain sample

Pipeline	Reads After Filtering	Total Correct Reads	Total Incorrect Reads	Accurate Reads (%)	Total *wzi* types
error0.005	13,367	13,338	29	99.78	8
error0	6,181	6,179	2	99.96	3

Supplemental Table 2: The *wzi*-Seq method has high read accuracy with 5 and 12 *wzi* type mixtures

Sample Mixture	Pipeline	Average Reads After Filtering	Average Total Correct Reads	Average Total Incorrect Reads	Average Accurate Reads (%)	Average Total *wzi* Types
5 *wzi* Types	error0.005	9,932	9,854	78	99.3	13
	error0	4,322	4,319	3	99.9	6
12 *wzi* Types	error0.005	97,095	96,296	799	99.2	60
	error0	47,263	47,064	198	99.6	41

bioRxiv preprint doi: https://doi.org/10.1101/2025.11.05.686704; this version posted November 13, 2025. The copyright holder for this preprint (which was not certified by peer review) is the author/funder, who has granted bioRxiv a license to display the preprint in perpetuity. It is made available under aCC-BY 4.0 International license.

Supplemental Table 3: Summary table showing high precision between replicates for the 12 *wzi* type mixture

		error0.005		error0	
***wzi* Type**	**Expected Abundance (%)**	**Mean Abundance (%)**	**Standard Deviation**	**Mean Abundance (%)**	**Standard Deviation**
22	7.79	7.88	0.46	8.07	0.64
27	7.65	7.97	0.14	7.97	0.07
37	7.08	7.88	0.47	7.69	0.43
53	11.68	8.29	0.34	8.39	0.32
84	9.01	5.93	0.2	6	0.32
151	5.39	6.32	0.39	6.1	0.36
252	6.38	8.02	1.16	7.03	1.71
266	10.55	6.8	0.06	7.17	0.26
333	7.74	10.24	0.25	10.62	0.41
349	6.89	9.48	0.29	9.56	0.31
426	9.8	12.13	0.81	12.31	0.84
522	10.04	8.29	0.13	8.55	0.32

Supplemental Table 4: Contrived 30 and 58 *wzi* type mixtures have decreased accuracy with the error0.005 pipeline

**Sample Mixture**	**Pipeline**	**Reads After Filtering**	**Total Correct Reads**	**Total Incorrect Reads**	**Accurate Reads (%)**	**Total *wzi* Types**	**Correct *wzi* Types**
30 *wzi* Types	error0.005	58200	55562	2638	95.47	99	29
	error0	27446	27296	150	99.46	56	28
58 *wzi* Types	error0.005	39822	38637	1185	97.02	114	53
	error0	18836	18744	92	99.51	76	53

Supplemental Table 5: Illumina Sequencing of Rectal Swab Plate Sweeps Validates *wzi*-Seq Results

**Sample**	**Rectal Swab Status**	**Rectal Swab *wzi* Types (>1%)**	**Plate Sweep Status (*wzi*-Seq)**	**Plate Sweep *wzi*-Seq *wzi* Types (> 1%)**	**Plate Sweep Status (Illumina)**	**Plate Sweep Illumina *wzi* Types**
16461	Mono	wzi_427	Mono	wzi_427	Mono	wzi_427
16969	Mixed	wzi_527, wzi_27	Failed	Failed	Failed	Failed
17000	Mixed	wzi_273, wzi_300, wzi_84	Mixed	wzi_273, wzi_300, wzi_84	Mixed	wzi_273, wzi_300, wzi_84
17117	Mono	wzi_537	Mono	wzi_537	Mono	wzi_537
17665	Mixed	wzi_360, wzi_537, wzi_9, wzi_108, wzi_223, wzi_227, wzi_340	Mixed	wzi_360, wzi_537, wzi_9, wzi_108, wzi_223, wzi_227, wzi_340, wzi_158		
18599	Mono	wzi_19	Mono	wzi_19	Mono	wzi_19
18801	Mono	wzi_173	Mono	wzi_173	Mono	wzi_173
18922	Mixed	wzi_379, wzi_53	Mixed	wzi_379, wzi_53	Mixed	wzi_379, wzi_53

bioRxiv preprint doi: https://doi.org/10.1101/2025.11.05.686704; this version posted November 13, 2025. The copyright holder for this preprint (which was not certified by peer review) is the author/funder, who has granted bioRxiv a license to display the preprint in perpetuity. It is made available under aCC-BY 4.0 International license.

613 Supplemental Table 6: Results from final multivariable model of gut mono-colonization vs. mixed
614 colonization and the risk of subsequent *Klebsiella* infection

Variable	Odds Ratio	95% CI (Lower - Upper)	P Value
**Age**	0.989	0.961 – 1.02	0.417
**Male Gender**	0.835	0.421 – 1.64	0.602
**Gut Colonization Month**[1]	1.02	0.955 – 1.08	0.608
**Mono-colonization**	2.18	1.08 – 4.57	0.034
**Prior Urinary Catheter**	1.92	0.947 – 4.00	0.0733
**Serum albumin ≥2.5 g/dL**	0.353	0.167 – 0.731	0.00546
**Serum albumin missing**	0.382	0.0688 – 1.82	0.234

[1]Colonization month was coded from 0-18 chronologically for each month that gut colonized subjects were recruited.

615

616

617
